# Human induced pluripotent stem cell-derived cardiomyocyte patches ameliorate right ventricular function in a rat pressure-overloaded right ventricle model

**DOI:** 10.1007/s10047-024-01479-3

**Published:** 2024-12-06

**Authors:** Takuji Watanabe, Takuji Kawamura, Akima Harada, Masaki Taira, Daisuke Yoshioka, Kazuo Shimamura, Tadashi Watabe, Eku Shimosegawa, Takayoshi Ueno, Shigeru Miyagawa

**Affiliations:** 1 Department of Cardiovascular Surgery, Osaka University Graduate School of Medicine, 2-2, Yamada-Oka, Suita, Osaka 565-0871 Japan; 2 Department of Nuclear Medicine and Tracer Kinetics, Osaka University Graduate School of Medicine, Osaka, Japan

**Keywords:** RV failure, Congenital heart disease, hiPS-CM transplantation, Pressure-overloaded RV failure, Cardiomyocyte patch

## Abstract

Right ventricular (RV) failure following surgical repair of congenital heart disease affects survival. Human induced pluripotent stem cell-derived cardiomyocyte (hiPS-CM) sheet transplantation ameliorated left ventricular dysfunction in preclinical studies, indicating its efficacy in RV failure in congenital heart disease. This study aimed to evaluate whether hiPS-CMs could improve RV function in rats with pressure-overloaded RV failure. F344/NJcl-rnu/rnu rats underwent pulmonary artery banding (PAB) via left thoracotomy. Four weeks after PAB, hiPS-CM patch transplantation to the RV was performed in the hiPS-CM group (n = 33), and a sham operation was performed in the sham group (n = 18). We evaluated cardiac catheterization, positron emission tomography data, and pathological results 8 weeks following PAB. RV end-diastolic pressure, the time constant of isovolumic relaxation, and end-diastolic pressure–volume relation were significantly ameliorated in the hiPS-CM group compared with in the sham group. Picrosirius red staining revealed that anti-fibrotic effects were significantly higher in the hiPS-CM group than in the sham group. Von Willebrand factor staining revealed significantly higher myocardial capillary vascular density in the hiPS-CM group than in the sham group. hiPS-CMs were detected in the epicardium 4 weeks after hiPS-CM sheet transplantation. The angiogenic gene expression in the myocardium was significantly higher in the hiPS-CM group than in the sham group. Overall, in rats with pressure-overloaded RV failure, hiPS-CM patch transplantation could improve diastolic function, suppress ventricular fibrosis, and increase capillary density, suggesting that it is a promising treatment for RV failure.

**Supplementary Information:**

The online version contains supplementary material available at 10.1007/s10047-024-01479-3.

## Introduction

Recent advancements in diagnostics, surgical techniques, and perioperative management have increased congenital heart disease (CHD) survival rates [1]. However, intracardiac repair often causes pressure-overloaded right ventricles (RVs) [2–4], which can lead to RV failure, contributing to mortality and morbidity. There are no effective therapies for RV failure except heart transplantation [5]. RV dysfunction in CHD is attributed to relative RV ischemia from chronic overloading and fibrosis [6, 7]; novel therapies must be established.

Recently, regenerative therapy using skeletal myoblasts [8] and induced pluripotent stem cell-derived cardiomyocytes (iPS-CMs) [9] has garnered attention to treat heart failure. We previously prepared cell sheets using temperature-responsive culture dishes [10, 11], developed a mass iPS-CM culture system, and reported the effectiveness of human iPSC (hiPS-CM) sheets in porcine ischemic cardiomyopathy [12].

The proposed mechanisms of the iPS-CM patch involve paracrine effects induced via angiogenic and antifibrotic factors. iPS-CM patch transplantation, with angiogenic and fibrosis-suppressing effects, may be effective for RV insufficiency. We herein hypothesized that hiPS-CMs would suppress or improve RV dysfunction caused by pressure overload by promoting angiogenesis and suppressing myocardial fibrosis. This study investigated whether hiPS-CM patches could improve RV function in RV pressure-overloaded rats.

## Materials and methods

### Study design

**Fig. 1:**
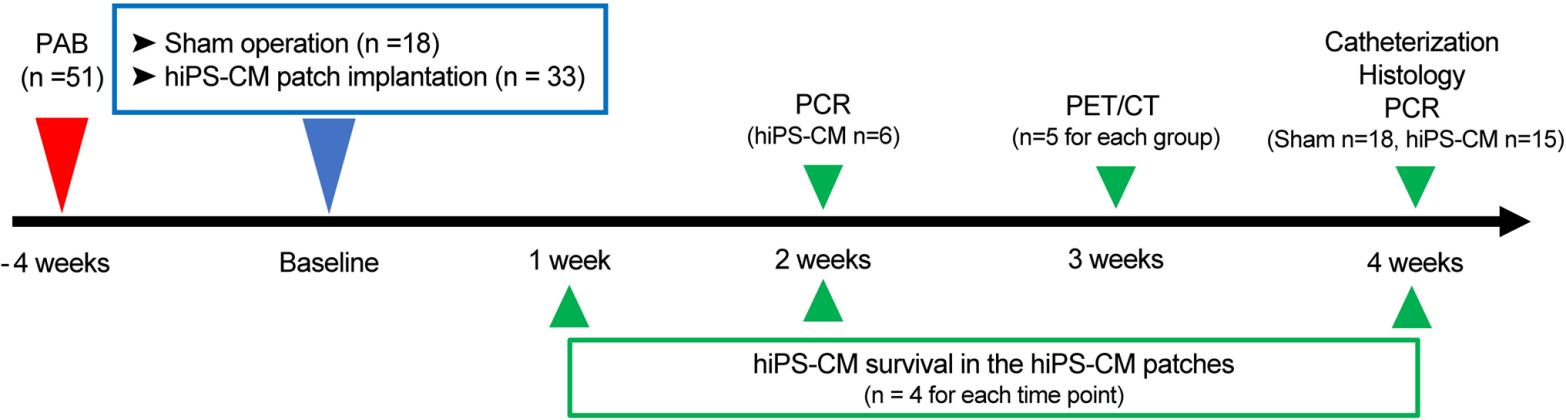
Study protocol. *PAB* Pulmonary artery banding; *hiPS-CM* Human induced pluripotent stem cell-derived cardiomyocyte, *PCR* Polymerase chain reaction, *PET/CT* Positron emission tomography/computed tomography

In this study, we evaluated the efficacy of hiPS-CM patches using a rat model of RV pressure overload. RV function was evaluated using positron emission tomography at 3 weeks and cardiac catheterization at 4 weeks following hiPS-CM patch transplantation or sham surgery; assessment of the transcript-level expression of proangiogenic cytokines in the RV myocardium was performed at 2 and 4 weeks following hiPS-CM patch transplantation and 4 weeks following sham surgery; and histological analysis was performed at 4 weeks following hiPS-CM patch transplantation or sham surgery (Fig. 1).

***Ethical approval***

The experimental protocols were approved by the Ethics Review Committee for Animal Experimentation of Osaka University Graduate School of Medicine (reference number 01–059-000). Institutional guidelines for the care and use of laboratory animals have been observed.

### Chronic pressure-overloaded RV model

Chronic RV pressure overload was induced in 7– 9 week-old male athymic nude rats (F344/NJcl-rnu/rnu; CLEA Japan, Tokyo, Japan) by permanently banding the main pulmonary artery (PA). Rats were anesthetized by isofluorane (2–3%, O_2_ carrier) inhalation, intubated, and placed on a volume-controlled respirator (2 mL, 60 cycles/min). The PA was dissected from the aorta through the fourth left intercostal space, and a 19-G needle was placed alongside the PA [13, 14]. Next, 3–0 polyester sutures were tied firmly around the needle and PA. The needle was removed to produce a fixed PA constriction proportional to the needle diameter (outer diameter: 1.1 mm).

### hiPS-CM patch transplantation

Four weeks after pulmonary artery banding, a second operation was performed through the fifth left intercostal space under general anesthesia. A prepared hiPS-CM patch (4 × 10^6^ cells/patch) was transplanted onto the anterior RV wall (hiPS-CM group, n = 33), or sham surgery was conducted (sham group, n = 18) (Supplementary Materials and Methods, Fig. S1). The hiPS-CM patch was fixed to the RV wall using Beriplast P (CSL Behring, King of Prussia, Pennsylvania, USA), and the pericardium was closed to prevent patch migration/adhesion to the chest wall. A control group of age-matched rats (n = 10) that did not undergo surgical intervention was included (Fig. 1).

### Histology

Myocyte size was determined using point-to-point perpendicular lines across the cross-sectional area of the cell through the nucleus (results are expressed as the average diameter of 10 myocytes that were randomly selected from five fields of each ventricle). The fibrotic area was calculated as the percentage of the myocardial area using Metamorph (Molecular Devices LLC, San Jose, CA, USA). Vessel density was quantified as the number of von Willebrand factor (vWF)-positive vessels per mm^2^. Data were obtained from five individual views of each heart.

### Statistical analyses

Statistical analyses were performed using JMP Pro (17.1.0; SAS Institute, Cary, NC, USA). Data are expressed as the mean ± standard deviation. Continuous variables were examined using Welch’s *t*-test, and normality was tested using the Shapiro–Wilk test. One-way analysis of variance was used to compare values among groups; when significant, group differences were compared using Tukey’s honest significance difference test. Relationships between capillary density and the percentage of myocardial fibrosis were assessed using linear regression and Pearson’s correlation. All *P*-values were two-tailed, and *P*-values < 0.05 were considered statistically significant.

## Results

### hiPS-CM patch transplantation improved RV function

**Fig. 2:**
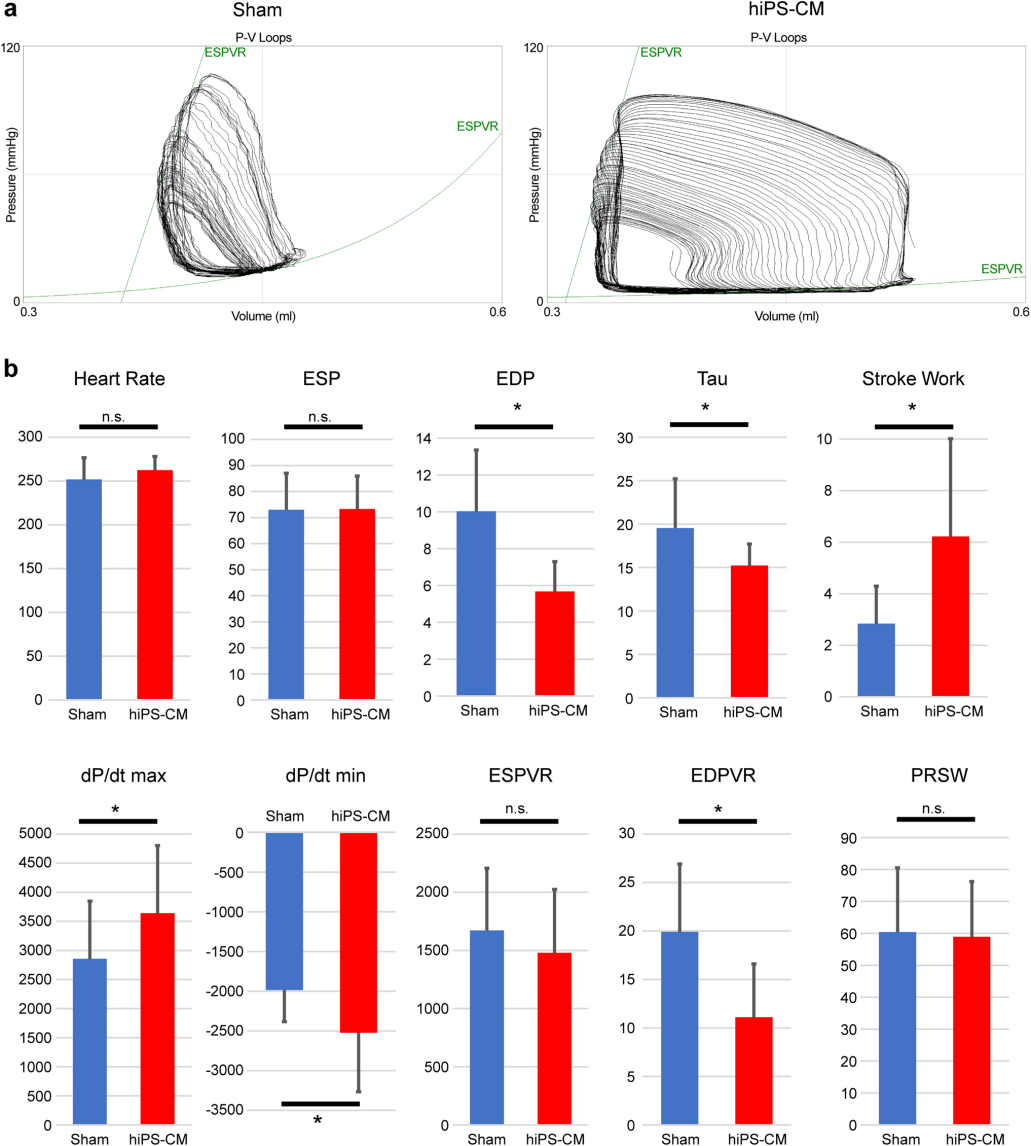
Hemodynamic indices during catheterization 4 weeks after the sham operation or hiPS-CM patch transplantation (sham, n = 18; hiPS-CM, n = 15). **a** Representative images of pressure–volume loops of the sham (left panel) and human induced pluripotent stem cell-derived cardiomyocyte (hiPS-CM) patch transplantation (right panel) groups under different loading conditions. The end-systolic pressure–volume relationship (ESPVR) is displayed as a straight line. The end-diastolic pressure–volume relationship (EDPVR) is displayed as a mono-exponential curve. **b** Comparison of basic hemodynamic indices and load-independent parameters analyzed by the pressure–volume loop. *P < 0.05. *ESP* End-systolic pressure, *EDP* End-diastolic pressure, *PRSW* Preload-recruitable stroke work, *n.s.* Non-significant

During cardiac catheterization, steady-state end-diastolic pressure (sham vs. hiPS-CM: 9.9 ± 3.5 vs. 5.8 ± 1.7 mmHg; *P* < 0.001) and tau (sham vs. hiPS-CM: 19.6 ± 5.9 vs. 15.3 ± 2.5 ms; *P* = 0.011), which are indicators of diastolic function, were significantly lower in the hiPS-CM group (Fig. 2b). The dP/dt max (sham vs. hiPS-CM: 2860 ± 981 vs. 3647 ± 1162 mmHg/s; *P* = 0.047) and dP/dt min (sham vs. hiPS-CM: -1973 ± 412 vs. -2508 ± 738 mmHg/s; *P* = 0.021), which are indicators of systolic and diastolic function, respectively, were significantly different between the groups (Fig. 2b). Stroke work (sham vs. hiPS-CM: 2.77 ± 1.51 vs. 6.29 ± 3.89 mmHg·mL; *P* = 0.0038) and cardiac output (CO) (sham vs. hiPS-CM: 13.7 ± 6.4 vs. 29.3 ± 17.2; *P* = 0.0039) were significantly higher in the hiPS-CM group (Fig. 2b). A typical example of a pressure–volume loop for each group is shown in Fig. 2a. In the pressure–volume loop analysis, the end-diastolic pressure–volume relation (sham vs. hiPS-CM: 19.9 ± 7.2 vs. 10.9 ± 5.6 /mL; *P* < 0.001), one of the best parameters reflecting diastolic function, was significantly lower in the hiPS-CM group (Fig. 2b). The end-systolic pressure–volume relation (sham vs. hiPS-CM: 1612 ± 637 vs. 1449 ± 544 mmHg/mL; *P* = 0.43) and preload recruitable stroke work (sham vs. c: 60.9 ± 20.5 vs. 59.0 ± 17.9 mmHg; *P* = 0.78), the best indicators of systolic function, were not significantly different between groups (Fig. 2b).

### hiPS-CM patch transplantation improved mechanical efficiency

**Fig. 3:**
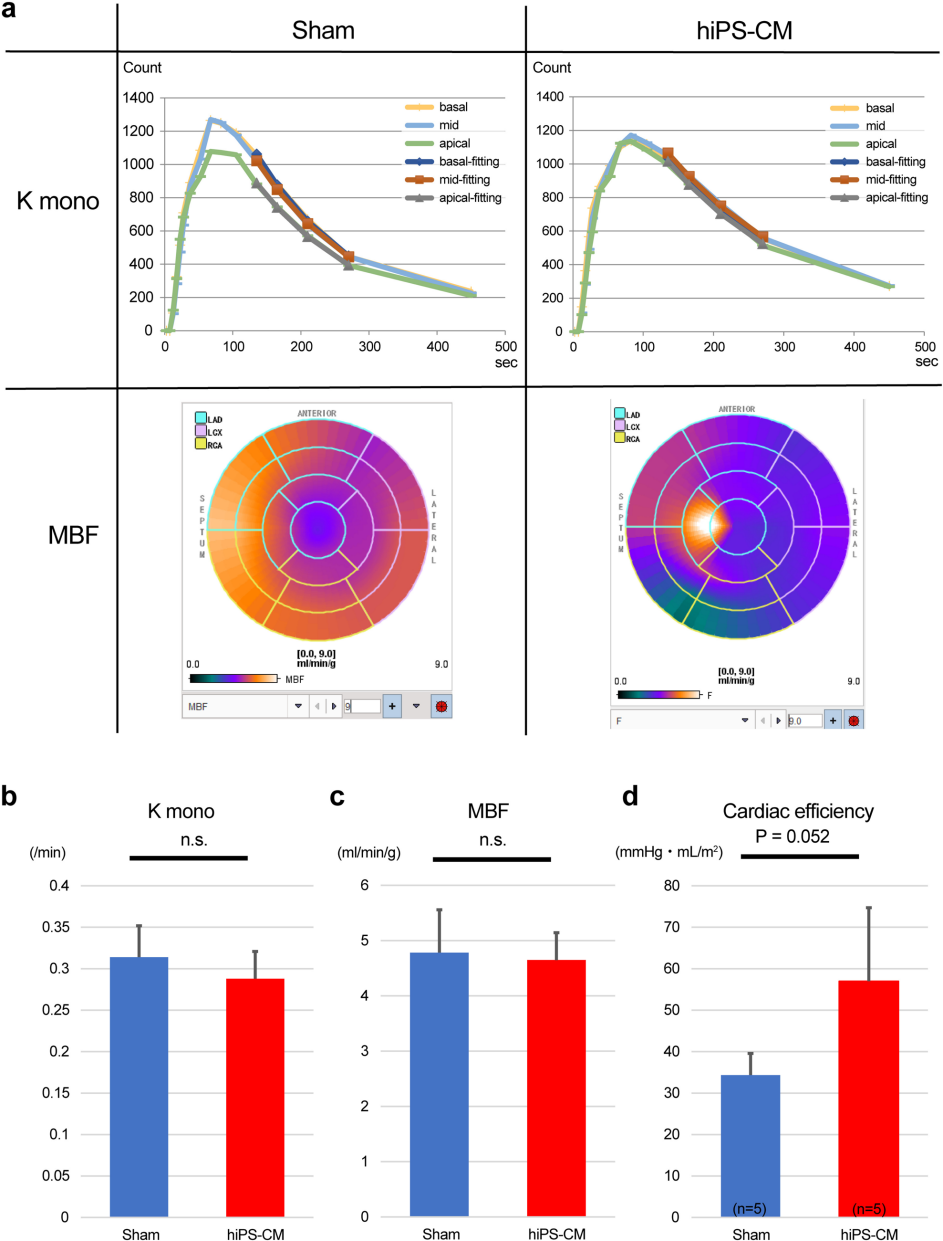
Measurement of in vivo myocardial oxidative metabolism based on the ^11^C-acetate clearance rate (sham, n = 5; hiPS-CM, n = 5). **a** Representative images of myocardial time-activity curves (upper panels) and the polar maps (lower panels) in the sham and human induced pluripotent stem cell-derived cardiomyocyte (hiPS-CM) patch transplantation groups. Kmono was calculated by mono-exponential fitting. **b** The acetate clearance rate (kmono) as a measure of right ventricular (RV) oxygen consumption. **c** Comparison of myocardial blood flow (MBF) in the RV. Kmono and MBF did not significantly differ between the sham and hiPS-CM groups. **d** The hiPS-CM group showed higher cardiac efficacy than the sham group. n.s., non-significant

Using positron emission tomography with ^11^C-acetate, kmono (sham vs. hiPS-CM: 0.31 ± 0.04 vs. 0.29 ± 0.03 /min; *P* = 0.28) and myocardial blood flow (sham vs. hiPS-CM: 4.78 ± 0.77 vs. 4.65 ± 0.50 mL/min/g; *P* = 0.75) did not significantly differ between groups (Fig. 3a–c). However, the cardiac efficiency (CE) approached significance (sham vs. hiPS-CM: 2130 ± 556 vs. 4692 ± 2115 mmHg·mL/m^2^; *P* = 0.052; Fig. 3d), suggesting a difference in correlation.

### Attenuation of RV hypertrophy due to angiogenesis and suppression of myocardial fibrosis after hiPS-CM patch transplantation

**Fig. 4:**
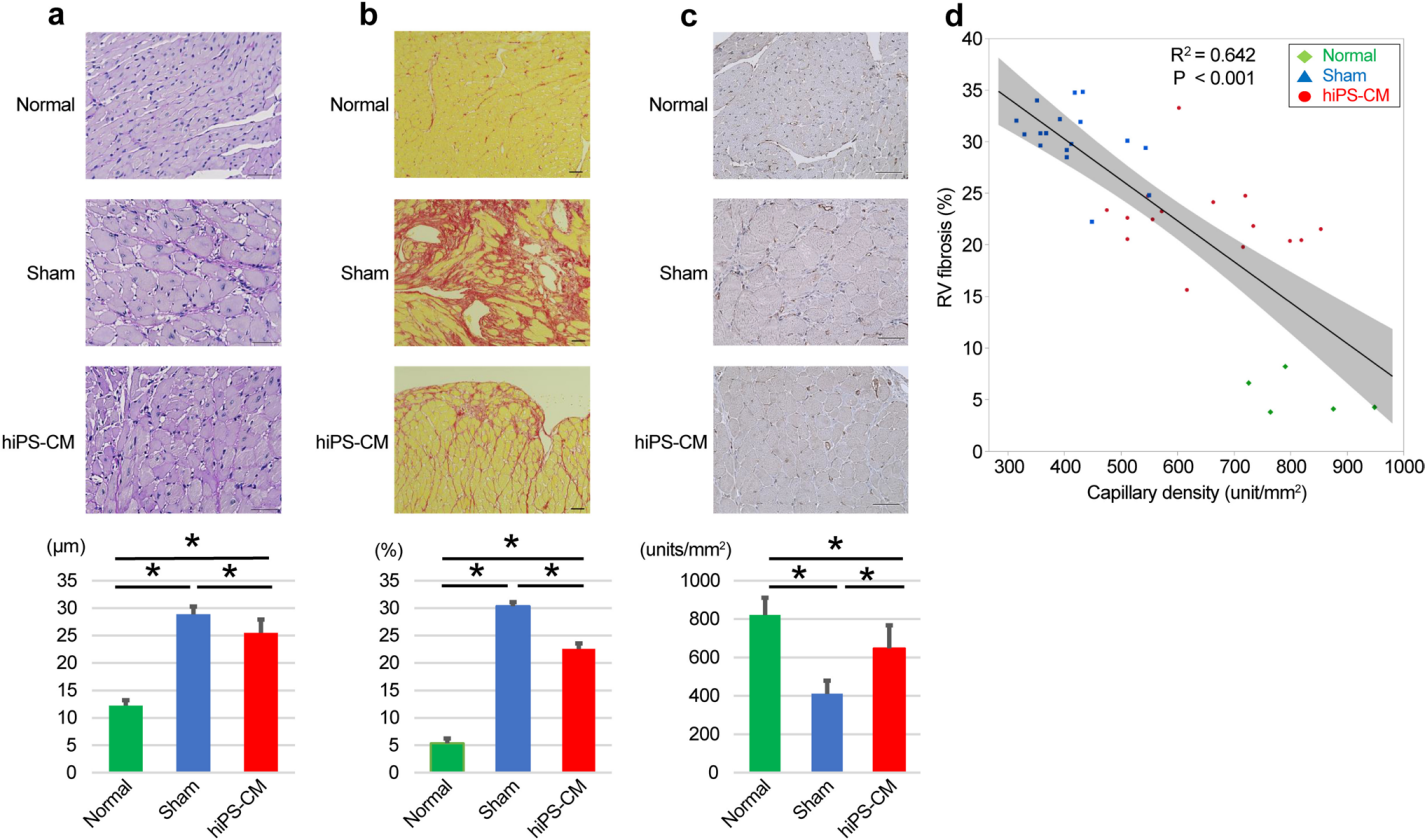
Histological assessment of hypertrophy, capillary density, and fibrosis in the right ventricle. **a** The left panels display representative images of periodic acid-Schiff-stained sections in each group. Scale bars: 50 μm. The right graph shows the size of cardiomyocytes in each group. **b** The left panels display representative images of myocardial fibrosis in each group, as assessed by Picrosirius Red staining. Scale bars: 50 μm. The right graph shows the percentage of myocardial fibrosis in each group. **c** The left panels display representative images of immunohistochemistry, assessed using the von Willebrand factor, in each group. Scale bars: 50 μm. The right graph shows the capillary density per unit area. **d** Capillary density was inversely correlated with the percentage of myocardial fibrosis in the right ventricle. *P < 0.05

Hematoxylin and eosin staining showed that the RV weight and RV hypertrophy decreased more in the hiPS-CM group than in the sham group (Fig. S2). According to the periodic acid-Schiff staining, the diameter of RV cardiomyocytes was significantly smaller in the hiPS-CM group than in the sham group (control vs. sham vs. hiPS-CM: 12.2 ± 0.98 vs. 28.9 ± 1.4 vs. 25.5 ± 2.4; all *P* < 0.001; Fig. 4a). Picrosirius red staining showed significantly more interstitial fibrosis within the RV myocardium in the sham and hiPS-CM groups than in the control group (control vs. sham vs. hiPS-CM: 5.4 ± 1.9% vs. 30.4 ± 3.1% vs. 22.6 ± 3.8%; *P* < 0.001 for sham and hiPS-CM vs. control; Fig. 4b). However, the myocardial RV fibrosis percentage was significantly lower in the hiPS-CM group than in the sham group (*P* < 0.001). Furthermore, the myocardial capillary density, assessed by vWF staining, was significantly higher in the hiPS-CM group than in the sham group (control vs. sham vs. hiPS-CM: 821 ± 90 vs. 411 ± 68 vs. 647 ± 121 units/mm^2^; *P* = 0.003 for sham vs. hiPS-CM; Fig. 4c). RV capillary density and myocardial fibrosis were inversely correlated (Fig. 4d).

### hiPS-CM engraftment after transplantation

**Fig. 5:**
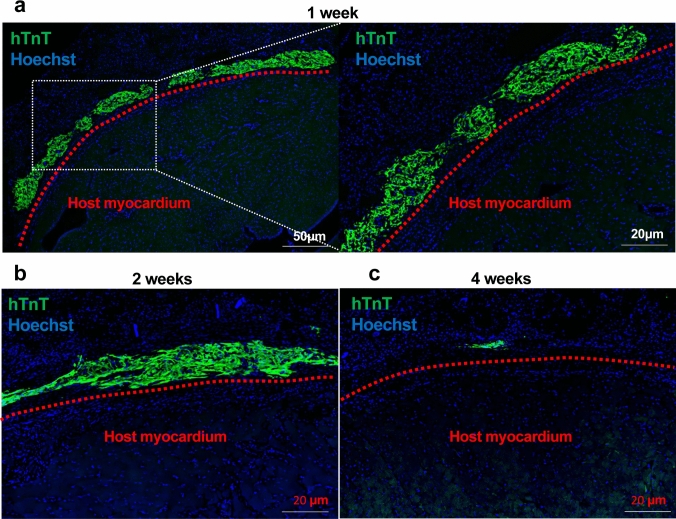
Serial changes after the engraftment of hiPS-CMs. Sections of the right ventricular myocardium 1, 2, and 4 weeks after hiPS-CM patch transplantation were immunolabeled with the anti-troponin T antibody, which reacted specifically with human troponin T (hTnT; green). Nuclei were stained with Hoechst 33,258 (blue). hiPS-CM, human induced pluripotent stem cell-derived cardiomyocyte

We investigated the degree of hiPS-CM engraftment using immunolabeled Sects. 1, 2, and 4 weeks after hiPS-CM patch transplantation (n = 4 each). hiPS-CMs were detected in the RV myocardium of rats even 4 weeks after transplantation, although there were clear reductions in levels compared with those observed at 1 and 2 weeks after hiPS-CM patch transplantation (Fig. 5).

### Upregulation of angiogenic cytokine expression after hiPS-CM patch transplantation

**Fig. 6:**
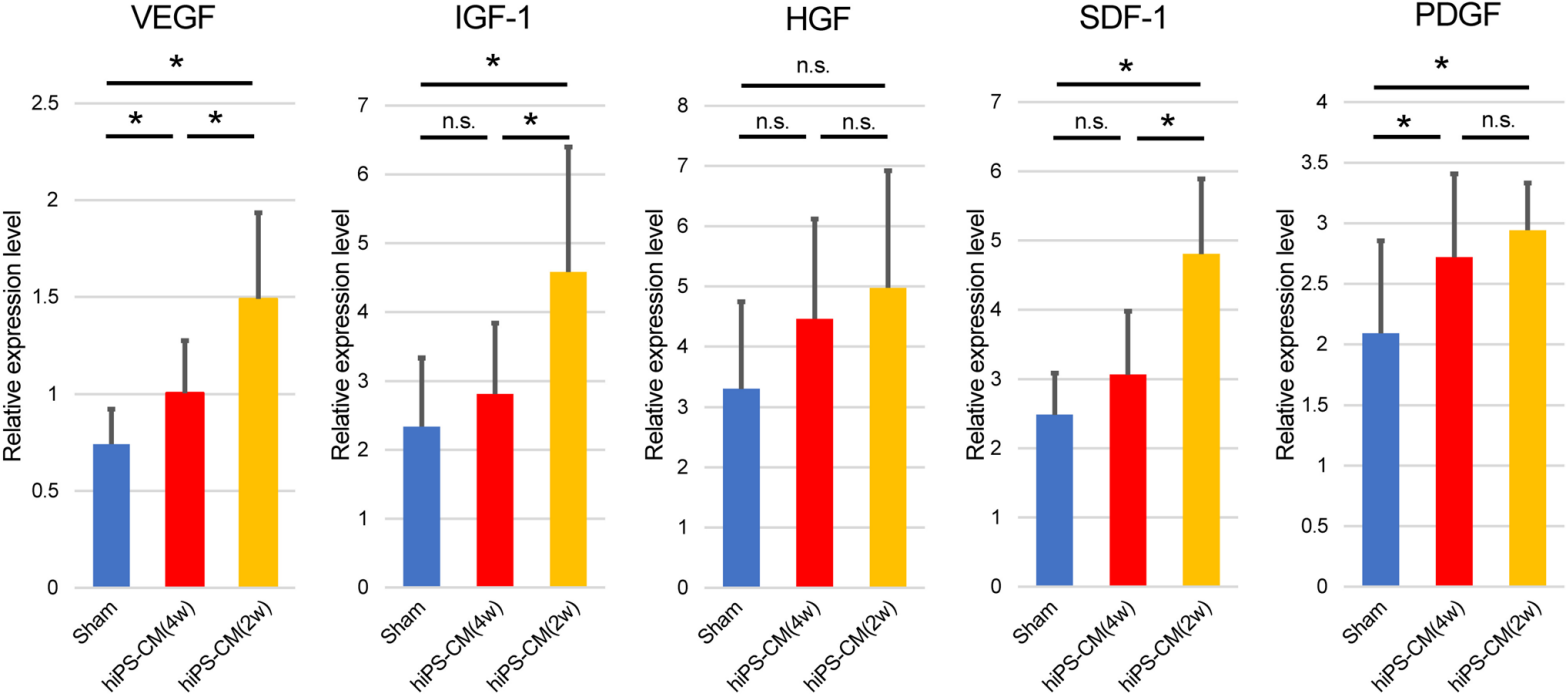
Quantitative polymerase chain reaction analysis of angiogenic cytokine-related gene expression in the right ventricular myocardium. *P < 0.05; *n.s*. Non-significant, *VEGF* Vascular endothelial growth factor, *IGF-1* Insulin-like growth factor 1, *HGF* Hepatocyte growth factor, *SDF-1* Stromal cell-derived factor 1, *PDGF* Platelet-derived growth factor

The expression of *vascular endothelial growth factor* (*VEGF*) (sham vs. hiPS-CM(4w): 0.74 ± 0.18 vs. 1.01 ± 0.27; *P* = 0.02) and *platelet-derived growth factor* (*PDGF*) (sham vs. hiPS-CM(4w): 2.09 ± 0.76 vs. 2.72 ± 0.69; *P* = 0.04) was significantly higher in the hiPS-CM(4w) group than in the sham group (sham, n = 18; hiPS-CM(4w), n = 15; Fig. 6). The expression of *insulin-like growth factor 1* (*IGF-1*) (sham vs. hiPS-CM(4w) vs. hiPS-CM(2w): 2.34 ± 1.00 vs. 2.81 ± 1.02 vs. 4.58 ± 1.81; sham vs. hiPS-CM(4w), *P* = 0.47; sham vs. hiPS-CM(2w), *P* < 0.001; hiPS-CM(4w) vs. hiPS-CM(2w), *P* = 0.009) and *stromal cell-derived factor 1* (*SDF-1*) (sham vs. hiPS-CM(4w) vs. iPS-CM (2w): 2.49 ± 0.60 vs. 3.07 ± 0.91 vs. 4.80 ± 1.09; sham vs. hiPS-CM(4w), *P* = 0.11 sham vs. hiPS-CM(2w), *P* < 0.001; hiPS-CM(4w) vs. hiPS-CM(2w), *P* < 0.001) was significantly higher in the hiPS-CM(2w) group than in the sham group, but no significant differences were noted between the sham and hiPS-CM(4w) groups. Further, no significant difference was noted in the expression of *hepatocyte growth factor* (*HGF*) between any groups (sham vs. hiPS-CM(4w) vs. iPS-CM (2w): 3.31 ± 1.44 vs. 4.46 ± 1.65 vs. 4.98 ± 1.94; sham vs. hiPS-CM(4w), *P* = 0.1; sham vs. hiPS-CM(2w), *P* = 0.08; hiPS-CM(4w) vs. hiPS-CM(2w), *P* = 0.79). However, the expression of all angiogenic factors was higher in the hiPS-CM(2w) group than in the hiPS-CM(4w) group.

## Discussion

This study demonstrated that hiPS-CM improves RV function in the RV pressure-overloaded rat model by promoting angiogenesis in the RV myocardium and suppressing myocardial fibrosis. The main findings are as follows: (1) Diastolic function was significantly improved; (2) fibrosis of the RV myocardium was suppressed, and the capillary density of the RV myocardium increased; and (3) the expression of angiogenesis-related factors was significantly increased. hiPS-CM transplantation has exhibited therapeutic efficacy in studies using left ventricular heart failure models [12, 15, 16]. The present study is the first to show the efficacy of hiPS-CMs for RV dysfunction caused by chronic pressure overload.

The pressure-overloaded RV first adapts by increasing its contractility by enhancing its intrinsic contractile properties and muscle hypertrophy to maintain CO [17, 18]. However, long-term pressure overload results in RV dilatation instead of hypertrophy. Subsequently, RV diastolic function gradually deteriorates, and systolic function is impaired, resulting in decompensated RV failure [18–21]. RV ischemia and fibrosis underly decompensated RV failure.

Capillary rarefaction occurs during the transition from RV hypertrophy to RV failure [22, 23]. In the pressure-overloaded RV model, right coronary artery flow is reduced by a systolic gradient (aortic systolic pressure–RV systolic pressure) and a decrease in the right coronary diastolic perfusion pressure (aortic diastolic pressure–RV diastolic pressure) [24]. This leads to a loss of RV capillaries (i.e., a decrease in capillary density), which accelerates RV dysfunction. In this study, capillary density was significantly lower in the sham group than in the control group, whereas it was significantly higher in the hiPS-CM group than in the sham group. This suggests that RV myocardium microcapillaries can be maintained or increased by promoting angiogenesis after hiPS-CM patch transplantation and that RV dysfunction can be changed. Increases in angiogenesis-related gene expression, such as *VEGF* and *SDF-1*, were observed in the myocardium 4 weeks after patch transplantation. Herein, xenotransplantation was performed, and it is unclear whether hiPS-CM-expressed angiogenic factors acted directly on the RV myocardium of rats. However, cell transplantation can upregulate various cardioprotective factors via “crosstalk” between transplanted cells and host cardiac tissues [25]. Therefore, hiPS-CM transplantation likely caused the increases in *VEGF* and *HGF* expression in the rats’ myocardium.

RV pressure overload initially increases high-pressure tolerance and enhances collagen formation to maintain the RV shape; however, accumulation of myocardial collagen causes maladaptive changes in the collagen network structure and extracellular matrix integrity, resulting in fibrotic tissue replacing lost cardiomyocytes. This study showed that the antifibrotic cytokine *HGF* was highly expressed in the hiPS-CM group, suggesting that fibrosis progression may have been suppressed. Ischemia also triggers the development of fibrosis in the pressure-overloaded heart as part of a reparative response [26]. This may further enhance susceptibility to chronic ischemia due to a reduced coronary flow reserve and impaired diastolic coronary flow [24]. Similarly, RV fibrosis and capillary density were correlated, suggesting that angiogenesis induced by hiPS-CM transplantation improved myocardial ischemia, suppressed fibrosis, and prevented the exacerbation of RV functional impairment.

Herein, residual cardiomyocytes were confirmed even 4 weeks after hiPS-CM patch transplantation. Considering the number of residual cells, we speculate that hiPS-CMs did not directly contribute to improving RV contractility. The effects may largely depend on paracrine signaling, causing cytokine-induced angiogenesis and antifibrosis.

There were no major differences in the cytokines released by iPS-CMs and skeletal myoblasts in our previous report [15] that demonstrated that hearts transplanted with iPS-CMs showed increased vasculogenesis and decreased apoptosis compared to other cell types. The reason for this disparity remains unclear; however, this study showed that high levels of angiopoietin families that significantly contribute to the maturation of blood were detected in hiPS-CM culture supernatants (Fig. S1d). This may be one of the factors leading to increased angiogenesis in iPS-CMs than in skeletal myoblasts; however, further studies are needed to investigate this.

Cardiac catheterization is more reliable than echocardiography because obtaining accurate measurements during echocardiography in rats is difficult, and load-independent indicators can be measured during catheterization [27]. We previously reported that left ventricular systolic recovery may depend on angiogenesis via the paracrine effect with myocardial blood flow in the peri-infarct zone in a porcine ischemic cardiomyopathy model due to the ligation of the left anterior-descending coronary artery [12, 15, 16]. In this study, the paracrine effect also improved the overall RV ischemia, suppressed fibrosis, and improved RV diastolic dysfunction. Notably, this study did not provide strong evidence that hiPS-CMs ameliorated systolic/diastolic dysfunction in pressure-overloaded RVs. However, CE was higher in the hiPS-CM group than in the sham group. CE can decline due to tricuspid regurgitation, septal bowing [28], asynchronous activation, and diastolic dysfunction [29]. Therefore, the improvement in diastolic dysfunction may have contributed to the observed increase in CE.

The ideal outcome is for the hiPSC-CM patch to contract/relax synchronously within the recipient heart and contribute to improving cardiac function [30]. However, successful regenerative therapy using hiPS-CMs requires better engraftment of hiPS-CMs within the recipient myocardium for an extended period to enhance the therapeutic efficacy. This can be achieved by immunosuppression [31], promoting angiogenesis using hiPS-CM patch transplantation with an omentum flap [16, 32], and using suitable myocardial tissue with appropriate cardiomyocyte maturity [33] and orientation [34]. Although we did not use immunosuppressants because this study was conducted on nude rats, the engraftment of hiPS-CMs decreased considerably after 2 months. Future studies should examine the extent of the mechanical contribution of the transplanted cardiomyocytes to the contractile force of the diseased heart.

## Limitations

In this study, it was difficult to detect serial changes in RV function during cardiac catheterization because it is invasive in small animals. However, the expression of angiogenesis-related factors was significantly higher at 2 weeks than at 4 weeks after patch transplantation, suggesting that hiPS-CMs may ameliorate RV function more at 2 weeks than at 4 weeks. Further studies with large animals are required to assess this.

## Conclusion

HiPS-CMs attenuated RV diastolic dysfunction in pressure-overloaded RV rats, primarily due to angiogenesis promotion in the myocardium and myocardial fibrosis suppression.

## Supplementary Information

Below is the link to the electronic supplementary material.

Supplementary file1 (DOCX 1288 KB)

## Data Availability

All relevant data are included within the manuscript and the Supporting Information files.
