# Supplementary material for: Human induced pluripotent stem cell-derived cardiomyocyte patches ameliorate right ventricular function in a rat pressure-overloaded right ventricle model
Source: J Artif Organs. 2024 Dec 6;28(2):234–43. doi: 10.1007/s10047-024-01479-3 (PMC12078445; doi:10.1007/s10047-024-01479-3)
Supplement: Supplementary file 1 — Supplementary file1 (DOCX 1288 KB) [file 10047_2024_1479_MOESM1_ESM.docx]

**Electronic Supplementary Material**

**Materials and Methods**

**Human induced pluripotent stem cell-derived cardiomyocyte (hiPS-CM) culture and cardiomyogenic differentiation**

Human induced pluripotent stem (hiPS) cells (QHJI14s04; Kyoto University, Kyoto, Japan), cultured on iMatrix511 (Nippi, Tokyo, Japan)-coated dishes in StemFit Ak03N (Ajinomoto, Tokyo, Japan), were used. Cardiomyogenic differentiation was induced as previously reported [1] with slight modifications, and cardiomyocytes were dissociated [2]. Dissociated hiPS-CMs were cultured in Dulbecco’s Modified Eagle Medium (Nacalai Tesque, Kyoto, Japan) containing 10% fetal bovine serum (Sigma-Aldrich, St. Louis, MO, USA) and brentuximab vedotin (ADCETRIS^TM^, Takeda, Osaka, Japan) to eliminate residual undifferentiated cells [3]. The cells were suspended in a cell banker and frozen.

**Flow cytometry**

Cells were fixed (Fixation and Permeabilization Solution; BD Biosciences, NJ, USA), labeled with an unconjugated mouse anti-cardiac isoform of troponin T (cTnT) (Santa Cruz Biotechnology, TX, USA), and incubated with AlexaFluor-488 goat anti-mouse secondary antibodies (Thermo Fisher Scientific, Waltham, MA, USA). Data were analyzed using FACS Canto II (BD Biosciences) and Flowjo software (Tree Star, OR, USA).

**Characterization of hiPS-CMs**

The cardiomyocyte purity of the final induced pluripotent stem cell-derived cardiomyocyte (iPS-CM) culture was approximately 80% (flow cytometric detection of cTnT; **Fig. S1a**). Immunostaining revealed that human iPS-CMs expressed cTnT and alpha-sarcomeric actinin (**Fig. S1c**). Several cytokines and growth factors potentially involved in cardiac repair were detected in the hiPS-CM culture supernatants (Bio-Plex Human Cytokine Assay; Bio-Rad, Hercules, CA, USA; **Fig. S1d**).

**hiPS-CM patch preparation**

Thawed hiPS-CMs (4×10^6^ cells) were plated onto temperature-responsive dishes (UpCell; CellSeed, Japan) in Dulbecco’s Modified Eagle Medium containing 20% fetal bovine serum and cultured at 37 °C under 5% CO_2_. After 72 h, cells were detached and formed scaffold-free hiPS-CM patches (**Fig. S1b**).

**Hemodynamic assessment**

Four weeks after transplantation or sham surgery, right ventricular (RV) function was evaluated during cardiac catheterization under general anesthesia and mechanical ventilation. A median sternotomy exposed the RV apex with minimal bleeding. A conductance catheter (Unique Medical Co, Tokyo, Japan) was inserted from the RV apex toward the pulmonary valve, and a Miller 1.4F pressure-tip catheter (SPR-671; Millar Instruments, Houston, TX, USA) was simultaneously inserted from the anterior wall of the RV. The conductance system and pressure transducer controller (Integral 3 [VPR-1002]; Unique Medical Co., Ltd.) were configured as reported previously [4], and conductance, pressure, and intracardiac electrocardiographic signals were analyzed (Integral 3 software; Unique Medical Co. Ltd.). Blood conductivity was measured using a small (0.1-mm) cuvette, and the parallel conductance volume was measured using a hypertonic saline dilution method to determine the absolute volume [5].

The heart rate (HR), RV end-systolic and -diastolic pressure, stroke volume, cardiac output, stroke work, maximal rates of positive/negative pressure change (dP/dt max and dP/dt min), and the isovolumic relaxation time constant (tau [τ]) were derived from steady-state measurements. The relationship between RV pressure and volume was determined by temporary compression of the inferior vena cava. This pressure-volume analysis calculated the end-systolic pressure-volume relationship, end-diastolic pressure-volume relationship, and preload recruitable stroke work.

**Positron emission tomography**

To evaluate the effects of hiPS-CMs on global and regional myocardial blood flow (MBF) and cardiac oxygen consumption in the RV, positron emission tomography (PET; dynamic 10-min measurements) was performed with a bolus injection of ^11^C-acetate (~30 MBq) 3 weeks after hiPS-CM patch transplantation (n=5) or sham operation (n=5) (Inveon PET/CT System, Siemens, Munich, Germany). Blood pressure and HR were monitored using a tail cuff (BP-98A-L, Softron, Brussels, Belgium). Regions of interest were semiautomatically placed on the RV myocardium using PMOD (v4.0; PMOD Technologies, Zurich, Switzerland) by flipping the image horizontally. MBF was calculated using the one-tissue compartment model. To determine the global cardiac oxygen consumption, the acetate clearance rate (myocardial oxidative consumption [kmono]) of each measurement was calculated using automated mono-exponential fitting of the initial linear portion of the time-activity curves. Cardiac efficiency (CE) was calculated as: CE=stroke volume × systolic blood pressure × HR/kmono [6].

**Histology**

Four weeks after the patch transplantation or sham operation, hearts were removed under isoflurane anesthesia (5%), and the ventricles were dissected, formalin-fixed, paraffin-embedded, and cut (2-µm sections). RV wall thickness was measured using at least three hematoxylin and eosin (H&E)-stained sections (middle RV portion), and picrosirius red staining was performed to assess myocardial fibrosis. The fibrotic region was calculated as a percentage of the myocardial area. Periodic acid-Schiff staining was performed to examine cardiomyocyte hypertrophy. Samples were examined using optical microscopy (BZ 9000; Keyence, Osaka, Japan), and quantitative morphometric analysis was performed using Metamorph (Molecular Devices LLC, San Jose, CA, USA).

**Immunohistochemistry**

Endothelial cells were labeled with rabbit polyclonal anti-von Willebrand factor antibodies (1:50; Thermo Fisher Scientific), visualized using secondary antibodies (AlexaFluor 488 or AlexaFluor 555; Molecular Probes, Eugene, OR, USA), and counterstained using Hoechst 33342 (Dojindo, Kumamoto, Japan).

The sections were also labeled immunohistochemically using rabbit polyclonal anti-TNT antibodies (1:200; Abcam, Cambridge, UK), conjugated with secondary antibodies (AlexaFluor 488 or AlexaFluor 555; Molecular Probes), and counterstained using Hoechst 33342 (Dojindo). The labeled sections were assessed to investigate the viability of hiPS-CMs using fluorescence microscopy (Keyence).

**Quantitative real-time polymerase chain reaction**

Total RNA was isolated from RV cardiac tissue 2 weeks (hiPS-CM[2w]) and 4 weeks after hiPS-CM patch transplantation (hiPS-CM[4w]) and 4 weeks after the sham operation (RNeasy Fibrous Tissue Mini Kit; Qiagen, Hilden, Germany) and reverse transcribed to cDNA (SuperScript III Reverse Transcription Kit; Thermo Fisher Scientific). Quantitative real-time polymerase chain reaction was performed (ViiA 7 RealTime PCR System; Thermo Fisher Scientific) using a TaqMan probe (Thermo Fisher Scientific). All data were normalized to *glyceraldehyde-3-phosphate dehydrogenase* (*GAPDH*) levels and evaluated using the 2^–ΔΔCt^ method. *Vascular endothelial growth factor* (*VEGF*; assay ID: Rn01511601_m1), *insulin-like growth factor 1* (*IGF-1*; assay ID: Rn00710306_m1), *hepatocyte growth factor* (*HGF*; assay ID: Rn00566673_m1), *stromal cell-derived factor 1* (*SDF-1*; assay ID: Rn00573260_m1), *platelet-derived growth factor* (*PDGF*; assay ID: Rn01502596_m1), and *GAPDH* (assay ID: Rn01775763_g1) primers were used.

**Attenuation of RV hypertrophy after hiPS-CM patch transplantation**

Myocardial weight analysis showed that whole heart/body weight (control vs. sham vs. hiPS-CM: 3.46±0.58 vs. 5.29±0.62 vs. 5.33±0.68 mg/g), RV/body weight (control vs. sham vs. hiPS-CM: 0.35±0.06 vs. 1.35±0.08 vs. 1.38±0.12 mg/g), RV/whole ventricle (control vs. sham vs. hiPS-CM: 0.14±0.02 vs. 0.37±0.02 vs. 0.33±0.05), and RV/(left ventricle [LV]+interventricular septum [IVS]) (control vs. sham vs. hiPS-CM: 0.16±0.02 vs. 0.60±0.05 vs. 0.49±0.11) weight ratios were significantly greater in the sham and hiPS-CM groups than in the control group (*P*<0.001 in the sham and hiPS-CM groups vs. control; **Fig. S2a**). Furthermore, the RV/whole ventricle (*P*=0.036) and RV/LV+IVS (*P*=0.035) weight ratios were significantly lower in the hiPS-CM group than in the sham group.

Whole-heart sections revealed thickened RV walls, enlarged cavities, and a shift of the IVS toward the left side in the sham and hiPS-CM groups (**Fig. S2b**). The RV wall was significantly thicker in the sham and hiPS-CM groups than in the control group (control vs. sham vs. hiPS-CM: 791±12 vs. 1669±259 vs. 1546±256 μm; *P<*0.001 in the sham and hiPS-CM groups vs. control); however, no significant difference was observed between the sham and hiPS-CM groups (*P*=0.32; **Fig. S2b**).

**References**

1. Ito E, Miyagawa S, Takeda M, Kawamura A, Harada A, Iseoka H, et al. Tumorigenicity assay essential for facilitating safety studies of hiPSC-derived cardiomyocytes for clinical application. Sci Rep 2019;9:1881.

2. Ito E, Miyagawa S, Yoshida Y, Sawa Y. Efficient method to dissociate induced pluripotent stem cell-derived cardiomyocyte aggregates into single cells. Methods Mol Biol 2021;2320:29–33.

3. Sougawa N, Miyagawa S, Fukushima S, Kawamura A, Yokoyama J, Ito E, et al. Immunologic targeting of CD30 eliminates tumourigenic human pluripotent stem cells, allowing safer clinical application of hiPSC-based cell therapy. Sci Rep 2018;8:3726.

4. Sato T, Shishido T, Kawada T, Miyano H, Miyashita H, Inagaki M, et al. ESPVR of in situ rat left ventricle shows contractility-dependent curvilinearity. Am J Physiol 1998;274:H1429–34.

5. Baan J, van der Velde ET, de Bruin HG, Smeenk GJ, Koops J, van Dijk AD, et al. Continuous measurement of left ventricular volume in animals and humans by conductance catheter. Circulation 1984;70:812–23.

6. Ishida M, Miyagawa S, Saito A, Fukushima S, Harada A, Ito E, et al. Transplantation of human-induced pluripotent stem cell-derived cardiomyocytes is superior to somatic stem cell therapy for restoring cardiac function and oxygen consumption in a porcine model of myocardial infarction. Transplantation 2019;103:291–8.

**Supplementary Figures**


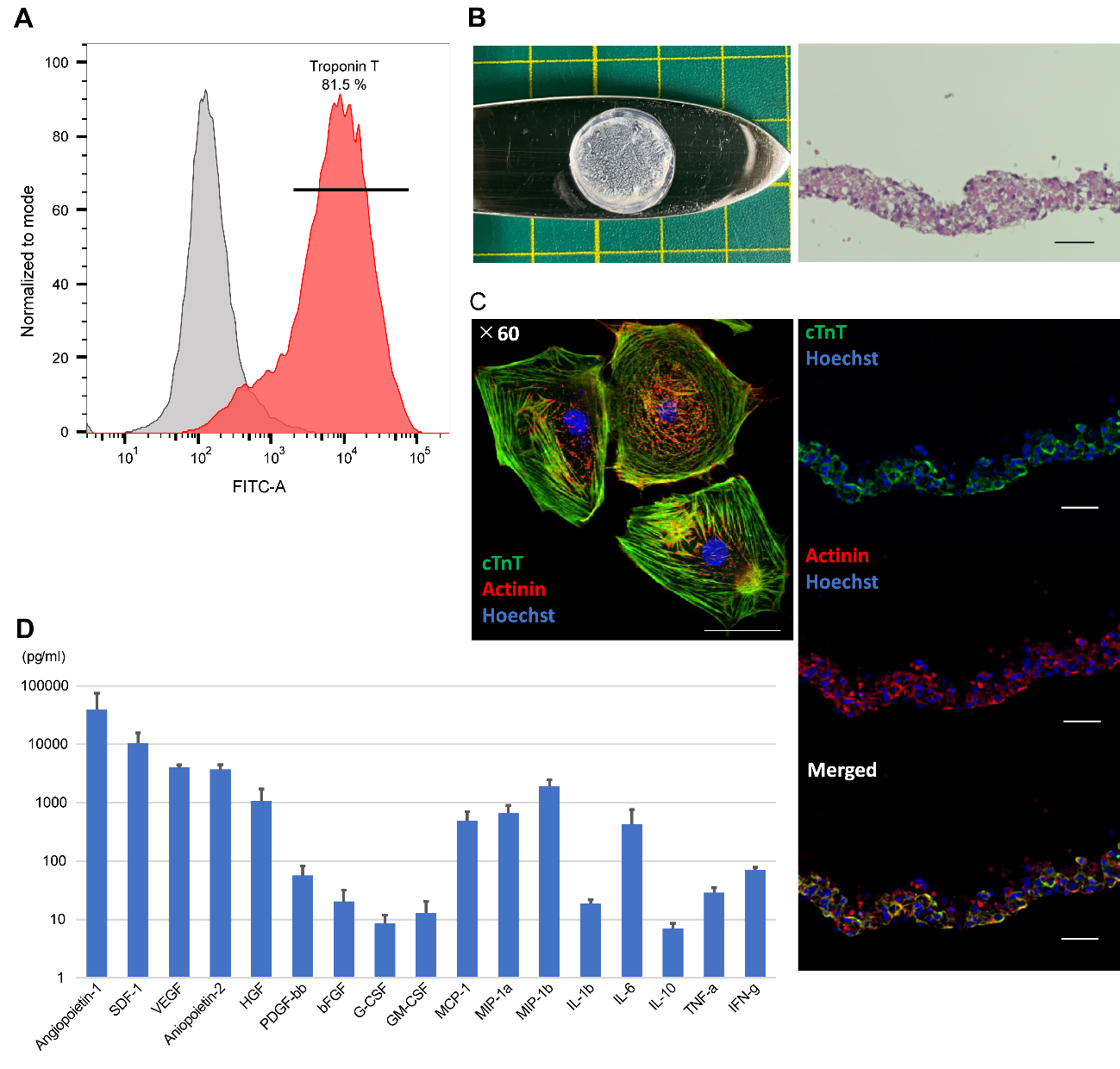


**Fig. S1** **Characterization of the human induced pluripotent stem cell-derived cardiomyocyte (hiPS-CM) patch**. (a) Expression of cardiac troponin T (cTNT) after differentiation and purification of hiPS-CMs by flow cytometry. (b) An hiPS-CM patch and hematoxylin and eosin (H&E) staining of an hiPS-CM patch. The diameter and thickness of the hiPS-CM patch were approximately 1.5 cm and 50 µm, respectively. Scale bar, 50 μm. (c) Immunostaining of hiPS-CMs and the hiPS-CM patch. hiPS-CMs were immunolabeled with anti-cTnT (green) and anti-alpha-sarcomeric actinin (α-actinin; red) antibodies and stained with Hoechst 33258 (blue). Scale bars, 50 μm. (d) In vitro screening for cytokines and growth factors. SDF-1, stromal cell-derived factor 1; IL, interleukin; VEGF, vascular endothelial growth factor; HGF, hepatocyte growth factor; TNF, tumor necrosis factor; IFN, interferon


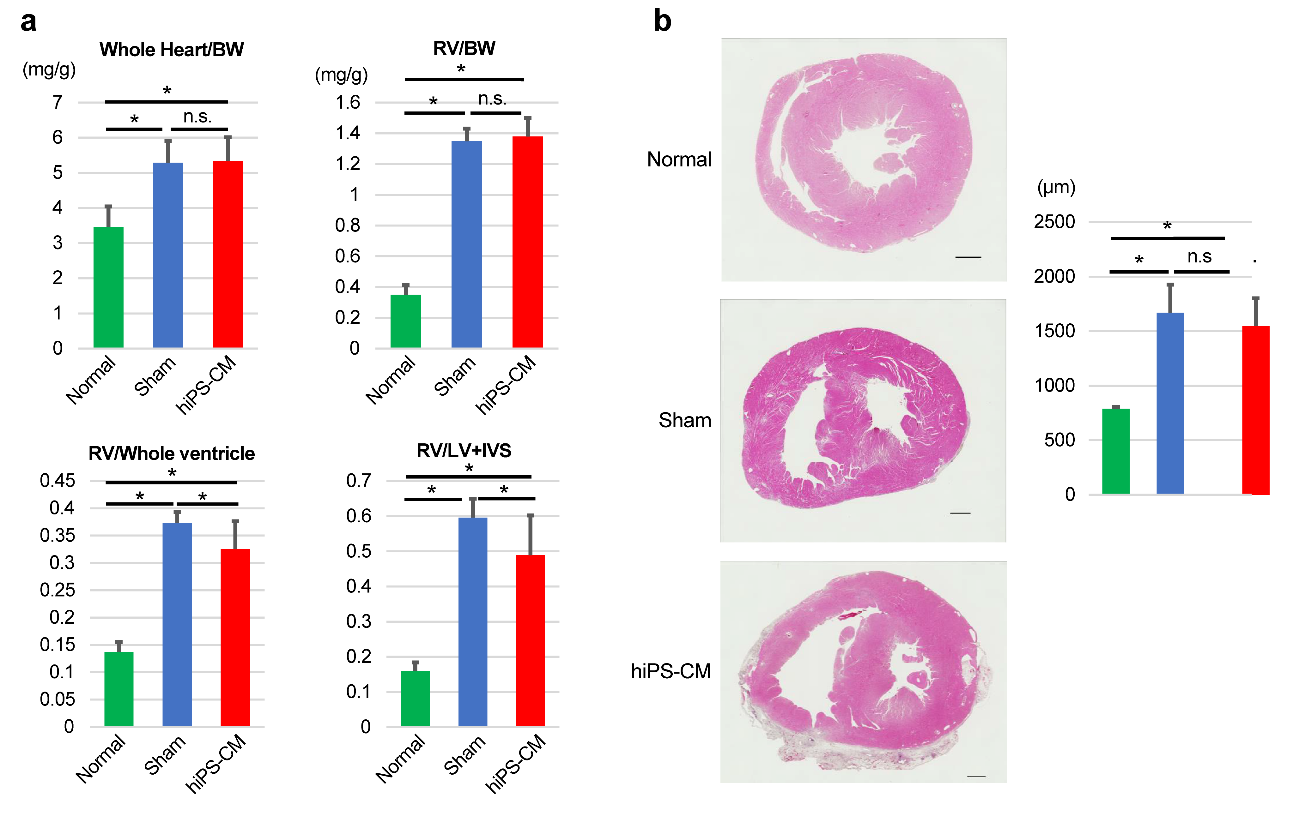


**Fig. S2 Weight analysis of right ventricular cardiomyocytes and histological assessment of RV hypertrophy**. (a) Weight analysis 4 weeks after the sham operation and hiPS-CM patch implantation. (b) The left panels display representative images of hematoxylin and eosin (H&E) stained sections in each group. Scale bars, 1000 μm. The right graph shows the RV wall thickness in each group. **P*<0.05. BW, body weight; RV, right ventricle; LV, left ventricle; IVS, interventricular septum; n.s., non-significant; hiPS-CM, human induced pluripotent stem cell-derived cardiomyocyte
